# Prehabilitative high-intensity interval training and resistance exercise in patients prior allogeneic stem cell transplantation

**DOI:** 10.1038/s41598-023-49420-7

**Published:** 2023-12-12

**Authors:** Rea Kuehl, Jule Feyer, Matthias Limbach, Antonia Pahl, Friederike Stoelzel, Heidrun Beck, Annika Wegner, Friederike Rosenberger, Peter Dreger, Thomas Luft, Joachim Wiskemann

**Affiliations:** 1 Working Group Exercise Oncology, Division of Medical Oncology, National Center for Tumor Diseases (NCT) Heidelberg, Im Neuenheimer Feld 460, 69120 Heidelberg, Germany; 2 Department of Hematology, Oncology and Stem Cell Transplantation, Medical Center -University of Freiburg, Faculty of Medicine, University of Freiburg, Freiburg, Germany; 3 Prevention Center of the National Center for Tumor Diseases Dresden (NCT/UCC), Dresden, Germany; 4 Department of Sports Medicine, University Center for Orthopedics, Trauma and Plastic Surgery, Dresden, Germany; 5 Medical Clinic II, Hematology-Oncology, University Clinic Frankfurt, University Cancer Center (UCT) Frankfurt, Frankfurt, Germany; 6 Division of Health Sciences, German University of Applied Sciences for Prevention and Health Management, Saarbrucken, Germany; 7 Department of Internal Medicine V, University Hospital Heidelberg, Heidelberg, Germany

**Keywords:** Haematological cancer, Cancer, Oncology, Cancer

## Abstract

Physical capacity prior allogeneic stem cell transplantation (allo-HCT) has been shown as a relevant prognostic factor for survival after transplant. Therefore, we evaluated feasibility and preliminary efficacy of a high-intensity interval training (HIIT) and moderate to high-intensity resistance exercise (RE) to increase physical capacity in patient’s prior allo-HCT. In this multicentre single arm pilot study, a supervised exercise program was performed twice weekly for 4–12 weeks prior allo-HCT, depending on the individual time remaining. Outcomes were feasibility (recruitment, adherence, safety), physical capacity (cardiorespiratory fitness [VO2peak], muscle strength) and patient reported outcomes (physical functioning, fatigue). Thirty patients were intended, 16 could be included, and 14 completed post intervention assessment (75% male, 55 ± 11 years). The study was stopped early due to a low recruitment rate. Nine patients (64%) reached the initial minimum planned number of eight exercise sessions. Individual adherence was high with 92% for HIIT and 85% for RE. 87% of all performed exercise sessions were completed without complaints and VO2peak increased significantly from 20.4 to 23.4 ml/kg/min. The low recruitment rate suggests that initiation of the intervention concept immediately before allo-HCT is feasible only in a small number of patients. In particular, the timeframe directly prior allo-HCT seems too short for exercise interventions, although the exercise program was designed to improve outcomes in a very short time frame. HIIT and RE were feasible, effective and well accepted by the included patients.

## Introduction

Allogeneic stem cell transplantation (allo-HCT) is an important treatment option for many hematological malignancies, e.g. leukemia or lymphoma. Although five-year survival rate is increasing due to advances in the management of complications e.g. graft versus host disease (GvHD) or infections^1,2^, physical capacity after allo-HCT remains low^3–5^, and patients suffer from reduced quality of life (QOL)^6,7^. Almost half of the patients show moderate to severe impairments in exercise capacity one year after allo-HCT resulting in disability, fatigue und reduced QOL^4^.

Evidence suggests that exercise interventions in patients with haematological cancer are safe and beneficial. Positive effects range from improved physical capacity to psychosocial effects, resulting in enhanced QOL^8–10^.

However, patients experience significant reductions in physical capacity^11–15^ already prior allo-HCT. First observational studies demonstrate that objectively assessed cardio-respiratory fitness (CRF) through a cardiopulmonary exercise test at exhaustion (CPET, with VO2peak outcome measure) was a significant predictor for overall survival and non-relapse mortality^16,17^. Retrospective data support these findings^18^.

Therefore, it seems to be important to implement exercise interventions prior allo-HCT to achieve a higher level of physical function prior treatment. This concept of prehabilitation has been already examined in cancer pre-surgical settings and proved to be safe and feasible^19–21^. However, only one small pilot study proved the concept of prehabilitation in allo-HCT. The intervention (individually tailored home-based exercise) was safe and feasible and after at least six weeks of intervention, patients significantly improve their CRF^22^.

In summary, the pre-transplant period seems to be an important and insufficiently investigated research area with the prospect to improve clinical outcomes and prognosis. However, the time frame from scheduling of planned allo-HCT to admission is usually relatively short. It thus seems pivotal to choose an exercise intervention that is able to improve physical capacity in a very short time such as high-intensity interval training (HIIT). HIIT is characterized by relatively short bouts of high-intensity workloads interspersed by periods of low-intensity workloads for recovery. Hereby, the total accumulated time of vigorous exercise is higher than what could be achieved during continuous exercise (usually moderate intensity)^23^. A recent meta-analysis of HIIT-effects in the prehabilitation of cancer treatment include seven RCTs and show a significant improvement in CRF and positive effects for health-related outcomes^24^.

Therefore, this pilot-study evaluates safety and feasibility of a combined moderate-to HIIT and RE program in patients with haematological cancer prior allo-HCT. Preliminary efficacy data on CRF, muscle strength, QOL and fatigue were also assessed.

## Methods

The study was a multicentre, prospective, single-arm study to investigate safety and feasibility of moderate to high-intensity HIIT and RE in patients with haematological cancer prior allo-HCT. The study (PRESENT-P trial [Pre-Exercise for allogeneic stem cell transplant patients: a pilot-study]) was conducted at the National Center for Tumor Diseases (NCT) Heidelberg and University Hospital Heidelberg, National Center for Tumor Diseases Dresden (NCT/UCC), the Department of Hematology, Oncology, and Stem Cell Transplantation University Medical Center Freiburg and the University Center for Tumor Diseases (UCT) Frankfurt, Germany, between November 2016 and February 2019. The study was approved by the ethics committee (leading committee) of the University Hospital Heidelberg and validated by the local committees of the clinical partners and is registered at ClinicalTrials.gov (NCT03080792; 15/03/2017). All participants provided written informed consent prior initiating any study activity.

Patients were eligible to participate when they met the following inclusion criteria: hematological cancer with allo-HCT scheduled within the next four to 12 weeks, ≥ 18 years of age, sufficient German language skills, willing/ able to train at the provided exercise facilities twice weekly. Exclusion criteria were: heart insufficiency > NYHA III or uncertain arrhythmia, uncontrolled hypertension, severe renal dysfunction (GFR < 30%, Creatinine > 3 mg/dl), insufficient hematological capacity (either haemoglobin value below 8 g/dl or thrombocytes below 30.000/ µL, reduced standing or walking ability, further comorbidities that preclude participation in the exercise program, and engaging in systematic intense exercise training (at least 1h twice weekly). Patients were screened and recruited 4–12 weeks prior admission for allo-HCT. When allo-HCT was scheduled, patients were informed about the study by their treating hematologist. Further information they received from the study personnel at each center.

### Intervention

The prehabilitation intervention comprised of individually tailored supervised exercise sessions twice weekly for 4 to 12 weeks. The total duration of the individual intervention length depended on the final scheduling of transplantation. The exercise program consisted of a supervised HIIT on a bicycle ergometer and RE.

The HIIT-protocol was individualized based on the peak power output (PPO) achieved in the cardiopulmonary exercise test (CPET) prior intervention (t0) and comprised eight one-minute high-intensity bouts at 90–100% PPO, interrupted by one-minute rest periods at 50% PPO. Each session started with a 5–10 min warm up at 50% PPO and ended with a 5-min cool-down at 50% PPO. The first two exercise sessions were familiarization sessions at slightly lower intensities of 70% PPO. The selection of exercise intensities was based on own findings where we defined exercise intensities in a sample of patients prior allo-HCT^25^ and developed based on findings in patients with chronic heart failure^26^.

HIIT was followed by a 30 min moderate to intense RE program that include four to six exercises targeting the whole body (legs, arms, shoulders, back, and chest) at resistance training machines or free weights. RE was performed at an intensity of 12 repetition maximum, based on a one repetition maximum test (1-RM, only for resistance training machines). The 1-RM was applied after one familiarization session according to approved methods^27^. Two sets of each exercise were performed (2 × 12 repetitions) at 60–80% of 1 RM.

All exercise sessions were supervised by exercise specialists and performed at one of the recruiting centres or at certified exercise facilities provided by the OnkoAktiv network. The OnkoActive network is a German network with which we link health-oriented exercise facilities to enable training close to patients home. The exercise program complies with the American College of Sports Medicine (ACSM) exercise guidelines for cancer survivors^28^. When patients were not able to exercise at this high intensity, exercise intensity was reduced to a tolerable intensity (Rating of perceived exertion (RPE) < 18,^29^). Exercise was contraindicated during fever and acute infections, when thrombocytes dropped below 15.000/µl and when haemoglobin values were < 8 g/dl. Exercise intensity was reduced to 60% PPO when thrombocytes dropped under 20.000/µl and for RE to 40% 1 RM.

### Measures

All participants underwent assessment prior intervention (t0) and post intervention/ prior admission for allo-HCT (t1). CRF was measured via CPET on an electronically braked cycle ergometer (Ergoselect 100; Ergoline, Bitz, Germany). We measured peak oxygen uptake (VO2peak) and PPO for exercise prescription. During the graded exercise test (starting with 2-min unloaded warm-up, the load was increased by 10 W every minute, starting at 20 W) respiratory gas exchange was measured continuously using a breath-by-breath system (Ergostik; Geratherm Respiratory, Bad Kissingen, Germany). Gas exchange data and HR were stationary time averaged over 30s. VO2peak and HRmax were considered as the highest 30-s average value during or immediately post exercise. For safety reasons, a 12-lead electrocardiogram (ECG) was recorded continuously. Patients were encouraged to spend maximal effort until voluntary exhaustion.

Additionally, all patients underwent a 6-min walk test according to published guidelines^30^. For isometric muscle strength assessment, hand grip strength (HGS) was assessed on both sides using JAMAR handgrip dynamometer (Patterson Medical, Warrenville, IL, USA). The test was performed in a sitting position with the elbow attached to the trunk and elbow angle of 90º. The best out of three attempts for both sides was analysed. Additionally, maximum voluntary isometric contraction (MVIC) was measured with IsoMed 2000-system (D&R Ferstl GmbH, Hemau, Germany) for knee extension, elbow flexion and hip flexion at both sides.

QOL was assessed with the validated questionnaire of the European Organisation for Research and Treatment of Cancer (EORTC QOL-C30)^31^ and fatigue with the Multidimensional Fatigue Inventory (MFI)^32^. Sociodemographic and medical information were partly obtained from medical records and partly self-reported. Shortly before and directly after each exercise sessions patients reported on actual symptoms, based on the Edmonton Symptom Assessment Scale (ESAS, VAS 0–10)^33^ and provide a general feedback of the exercise session. The satisfaction of the patients with the intervention was assessed at the end of the intervention on a visual analogue scale (VAS 1–7).

### Statistical methods

Since PRESENT-P is a pilot-study that will provide information for a larger confirmatory RCT, no power calculation has been conducted and data were mainly of descriptive nature. We planned to recruit n = 30 patients in four centres. Due to the short time period between indication of allo-HCT and the start of treatment, our feasibility goal was to enable all included patients to perform at least eight prehabilitative exercise sessions before admission for transplantation. Further, we calculated exercise adherence for each patient individually, based on the individual number of available weeks. For this feasibility outcome, we considered a rate of 75% (at least eight exercise sessions possible and adherence to the sessions) as adequate. Regarding safety considerations, we reported on exercise related side effects (e.g. pain, bleeding signs). For preliminary efficacy, we did pre-post comparisons using Wilcoxon rank-sum test, Cohen’s d for VO2peak and present individual responses. All analyses were conducted in SPSS statistical software package (V26).

### Ethical approval

This study was performed in line with the principles of Declaration of Helsinki. Approval was granted by the Ethics Committee of the University Hospital Heidelberg (S-030/2016).

## Results

During the recruitment period between 11/2016 until 02/2019, 16 patients were included (n = 10 Heidelberg; n = 3 Dresden; n = 2 Freiburg, n = 1 Frankfurt). Enrolment stopped before the anticipated number of participants was reached because of low recruitment rate due to various reasons. Two patients dropped out (n = 1 allo-HCT postponed, n = 1 reason unknown). In total, 16 patients underwent baseline testing, n = 14 patients post-intervention testing (attrition rate 87.5%). Patient characteristics are presented in Table 1.

**Table 1:** Sociodemographic and medical data (n = 16).

	n		
Gender			
Male	12	75%	
Female	4	25%	
Age (mean, SD)	55.3	10.8	Range: 28–69
BMI (mean, SD)	25.0	5.4	Range: 18.6–42.8
Family status			
Living with partner(s)	11	69%	
Living alone	4	25%	
Missing	1	6%	
Education			
Low	4	25%	
Medium	2	13%	
High	9	56%	
Missing	1	6%	
Employment			
Yes	5	31%	
Up till diagnosis	8	50%	
Retired	1	6%	
Missing	2	13%	
Diagnosis			
MDS	5	31%	
Hodgkin’s/Non-Hodgkin’s Lymphoma	5	31%	
ALL	3	19%	
AML	2	13%	
AA	1	6%	
Time since Diagnosis in months (mean, SD)	44.7	58.3	Range: 1–183
First year after diagnosis	7	44%	
Therapy			
No	5	31%	
Chemotherapy	9	56%	
Radiation	3	19%	
Immunotherapy	4	25%	
Missing	1	6%	
Hemoglobin level in g/dl (mean, SD)	13.2	9.99	Range: 6.6–13.2
Missing	1		
Thrombocytes in /µl (mean, SD)	131	122	Range: 25–493
Missing	1		
Physical activity in the last 12 month before diagnosis (e.g. brisk walking, exercise activities)			
Yes	8	50%	
No	7	44%	
Missing	1	6%	
Physical activity in the last 4 weeks (e.g. brisk walking, exercise activities)			
Yes	4	25%	
No	7	44%	
Missing	1	6%	

### Feasibility, adherence and safety

**Figure 1:**
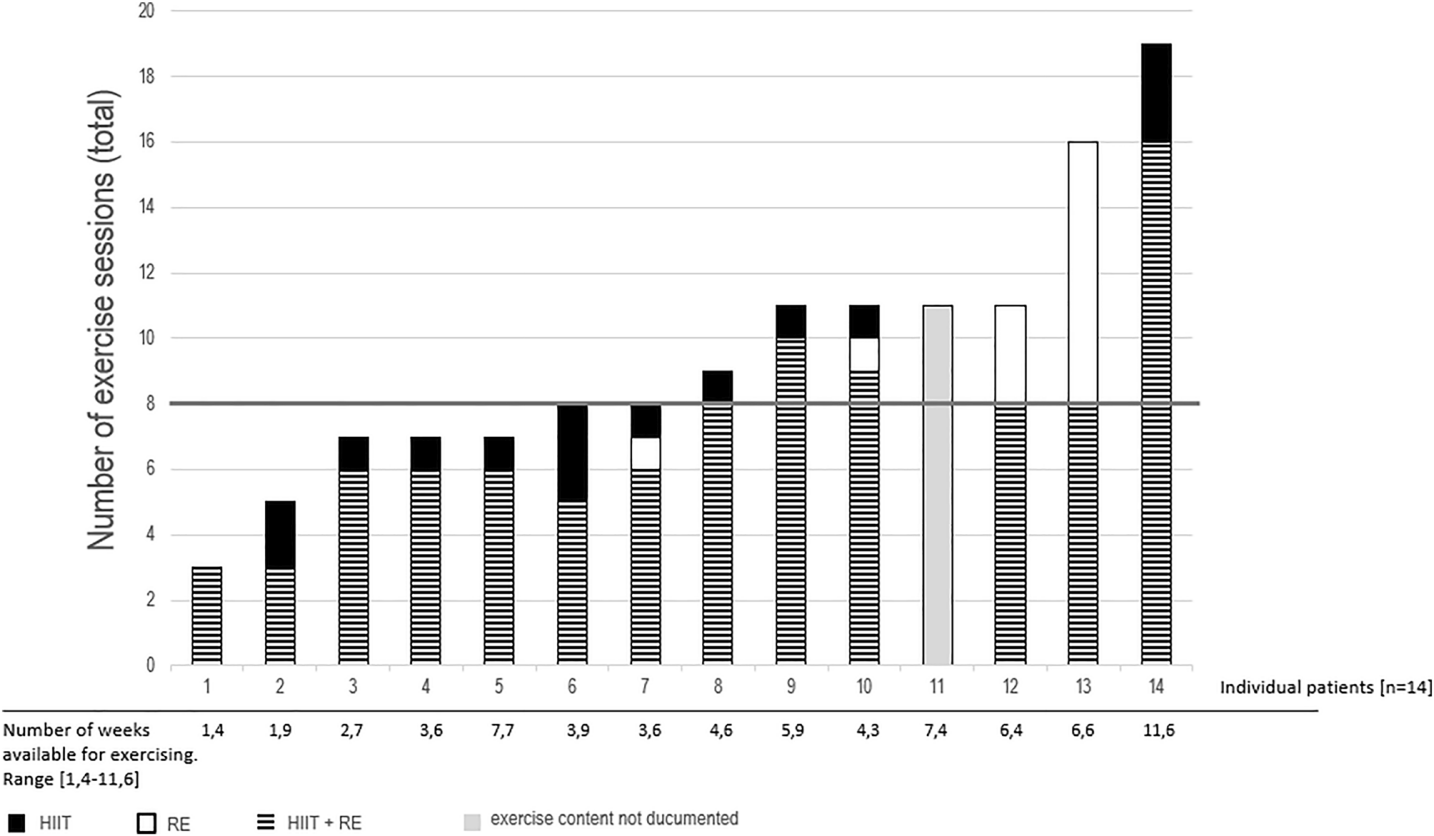
Exercise adherence. Individual amount of exercise sessions categorized in HIIT and RE (133 sessions in total). HIIT, high intensity interval training; RE, resistance exercise. [Example: Patient no 5 exercised 7 sessions in total, 6 sessions were combined sessions (HIIT + RE) and one session only HIIT].

In total, 133 exercise sessions were performed. Intervention length was 5.1 weeks (median 4.4, ranging 1.4–11.6 weeks). Nine patients (64.3%) reached the initial minimum planned number of eight exercise sessions. The mean number of completed exercise sessions was 9.5 (median 8.5, range 3–19). Figure 1 displays the individual adherence to HIIT and RE. Adherence to HIIT was 92% with overall mean interval per exercise of 7.4 from 8 intervals. Adherence to RE was 85.4% with overall mean of 11.7 repetitions (from 12 repetitions per 2 sets).

We received direct feedback of 128 exercise sessions. One-hundred-twelve exercise sessions (87.5%) were without complaints. Session with complaints include the following: four (3.1%) sessions had a premature ending (2 × during HIIT because of exhaustion and palpitations; 2 × during RE because of shoulder pain and lack of energy/weakness; in five (3.9%) sessions patients were unable to perform certain exercises (e.g. reduced intensity or termination of HIIT, termination of certain RE exercises; in seven (5.5%) sessions patients felt discomfort (e.g. 2 × vertigo directly after strain, 2 × exhaustion). At the beginning of 8 sessions (6.3%) patients reported on pain (e.g. muscle soreness, knee pain) associated with the last training session. Further reported complaints like dyspnoea, nausea, infections and bleeding signs were not related to exercise. No adverse events were reported. One-hundred-twelve (87.5%) sessions were rated with “just the right intensity”, eight sessions (6.3%) were rated with “overstraining” (5 × same patient) and seven sessions (5.5%) were rated with “underdemanding”. Enjoyment after each session was rated with 6.4 (MW, median 7) out of 7 points.

After intervention program, likeability (6.64), helpfulness (6.79), enjoyment (6.64), and recommendation to others (6.79) were rated on average with 6.65 out of 7 points. Patients rated the positive effect on the individual physical condition with 6.2 and the positive effect for the psychological effect with 5.4 out of 7.

### Preliminary efficacy

**Figure 2:**
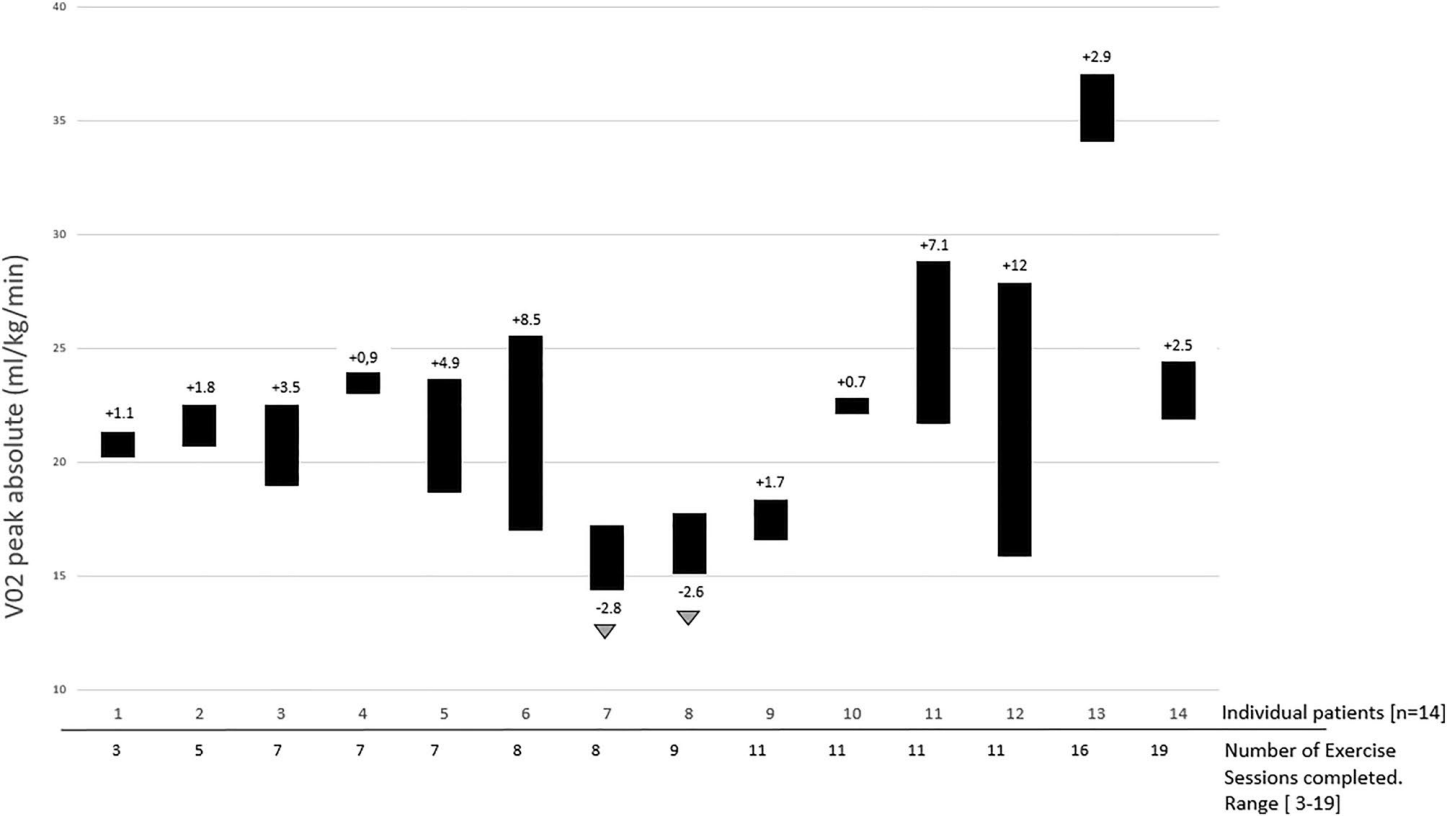
Individual change of VO2peak including individual baseline level and individual increase in ml/kg/min.

After the intervention program, VO2peak increased significantly from 20.4 to 23.4 ml/kg/min (median improvement 2.15 ml/kg/min, z = − 2.34, *p* = 0.019, CI 95% 0.67; 5.35, d = 1.6). The individual changes of VO2peak in ml/kg/min are presented in Fig. 2, including individual baseline level of all patients. PPO increased significantly from 117 to 131.2 W (median improvement 15 W, z = − 2.56, *p* = 0.010, CI 95% 5.04; 23.53).

Completed distance in the 6-min walk test increased significantly from 558.8 to 608.6 m (median improvement 30.5 m, z = − 2.03, *p* = 0.043). HGS (n = 9) increased significantly from 37.8 to 39.6 kg at the dominant side (median improvement 2 kg, z = − 2.06, *p* = 0.040) and from 35.4 to 39.5kg at the non-dominant side (median improvement 2 kg, z = − 2.38, *p* = 0.018). The MVIC test (n = 11) showed no significant changes in muscle strength of knee-extension (change of the dominant side: + 11.25 Nm), elbow-flexion (change of the dominant side: + 2.13 Nm) and the hip-flexion (change of the dominant side: − 14.00 Nm) at the dominant and non-dominant side.

Table 2 displays changes in patient reported outcomes (PROs). Significant improvements could be shown in physical functioning, role functioning, emotional functioning, fatigue and dyspnoea. Results of the MFI show a significant reduction of physical fatigue and reduced activity after intervention period.

**Table 2:** Effects of 4–12 week exercise intervention on Quality of Life (EORTC-C30) and Fatigue (MFI, different domains) (n = 14).

	T0	T1		
	MW(SD)	MW(SD)	Mean Change (CI 95%)	p*
EORTC-C30				
Global QOL	56.25 (21.65)	64.74 (14.89)	8.33 (− 2.73; 19.39)	0.107
Physical functioning	75.90 (15.04)	87.18 (11.69)	11.28 (2.83; 19.73)	**0.01**
Role functioning	52.56 (33.23)	71.79 (24.89)	34.62 (15.30; 53.93)	**0.041**
Emotional functioning	47.44 (25.54)	66.03 (20.26)	18.59 (10.59; 26.59)	**0.004**
Cognitive functioning	64.74 (14.89)	71.79 (18.49)	7.69 (− 3.65; 19.04)	0.161
Social functioning	56.41 (30.08)	64.10 (27.93)	7.69 (− 4.38; 19.76)	0.193
Fatigue	53.85 (19.27)	32.91 (17.34)	− 20.94 (− 33.59; − 8.29)	**0.007**
Nausea/vomiting	3.85 (7.31)	1.28 (4.62)	− 2.56 (− 6.35; 1.22)	0.157
Pain	23.08 (26.82)	19.23 (19.06)	− 3.84 (− 21.37; 13.67)	0.589
Dyspnoea	35.90 (31.80)	12.82 (16.88)	− 23.08 (− 42.16; − 3.40)	**0.024**
Insomnia	33.33 (36.00)	23.08 (25.04)	− 10.26 (− 25.39; 4.87)	0.157
Appetite loss	23.08 (25.04)	5.13 (12.52)	− 17.95 (− 35.62; − 0.28)	0.053
Constipation	12.82 (28.99)	7.69 (14.62)	− 5.13 (− 16.30; 6.05)	0.317
Diarrhoea	15.38 (25.87)	7.69 (19.97)	− 7.69 (− 22.30; 6.91)	0.257
Financial difficulties	25.64 (24.17)	25.64 (30.89)	0.00 (− 11.63; 11.63)	1
MFI (n = 13)				
General fatigue	13.31 (2.63)	12.00 (3.14)	− 1.31 (− 3.01; 0.39)	0.179
Physical fatigue	13.54 (4.58)	10.77 (3.59)	− 2.77 (− 5.15; − 0.39)	**0.03**
Reduced activity	12.54 (3.38)	9.85 (3.11)	− 2.69 (− 4.85; − 0.54)	**0.013**
Reduced motivation	8.31 (2.32)	7.62 (2.79)	− 0.69 (− 2.54; 1.16)	0.477
Mental fatigue	11.08 (4.82)	10.46 (4.24)	− 0.62 (− 2.26; 1.03)	0.453

Significant values are in [bold].

To assess a *single session effect* we compared the results from the ESAS Scale from directly prior to directly after every session. Patients had a significant reduction of pain (z = − 2.19, *p* = 0.028), anxiety (z = − 3.07, *p* = 0.002) and general well-being (z = − 2.82, *p* = 0.0.005 [lower values belongs to better general well-being]) directly after the exercise sessions. For nausea, depression, drowsiness, appetite and dyspnoea no changes could be found.

## Discussion

In this pilot-trial, we investigated a supervised HIIT and RE exercise program in patients prior allo-HCT with the aim to improve CRF and muscle strength in a very short timeframe. Results show that our prehabilitation exercise program was feasible, safe, effective, and well accepted by participating patients. However, due to low recruitment rate, the study was stopped before we reached the anticipated number of n = 30 patients.

The initial minimum planned number of eight exercise sessions prior allo-HCT was achieved by nine patients (64.3%). However, only 5.1 (range 1.4–11.6) weeks of training were possible due to scheduling for allo-HCT. Hence, it seems that adherence to exercise was not problematic (adherence depending on individual intervention length was 92% for HIIT and 85.4% for RE), but timing of the intervention, respectively the short time-period available after scheduling for allo-HCT.

All study centres had problems recruiting the patients within our planned recruitment period due to the tight time frame of the patients directly prior allo-HCT. For example, in Heidelberg (Germany) the recruiting study personnel attended the weekly transplant board to screen for eligible patients. Almost all patients received their schedule for allo-HCT only 2 weeks in advanced and were therefore not eligible for this study (inclusion criteria: 4–12 weeks prior planned admission/conditioning for allo-HCT). Main reasons for the short-term planning were the changing clinical status of patients and donor availability. However, this is an organisational problem which cannot be changed by the given procedures in the participating transplant clinics. Further challenges included personal resources, e.g. lack of work force and no capacity by physicians to screen for eligible patients. Therefore, recruitment was very challenging, and for this prehabilitation study concept is was not feasible. We thus stopped recruitment before we reached the anticipated number of n = 30 patients due to the longer recruitment period than anticipated and lack of manpower. Thus, a better collaboration between clinicians and exercise professionals should be sought for future projects.

Another, recently published pilot RCT in patients prior allo-HCT from the US faced similar problems (including insufficient time for exercise before allo-HCT) and the study was stopped because of low recruitment rate as well^34^. Given these similar implementation barriers of studies in this setting it needs to be questioned, whether the chosen approaches fit with the clinical patient flow as wells as with the demands of allo-HCT patients and their medical treaters. Scientifically, it seems to be indisputable that patients should be at their individual best physical constitution and function level, when they are entering the transplant process. If the medical team is also convinced by this evidence, there is a need to offer an extend time frame to enrol patients in a prehabilitation program prior allo-HCT which requires a modified transplant preparation procedure. Additionally, by incorporating exercise prehabilitation as a routine procedure in the allo-HCT preparation this might led to improved transplant eligibility. Facing the older and partly multimorbid/frailpatient populations scheduled for allo-HCT, future comprehensive transplant programs will not be able to ignore the potential of prehabilitation measures when preparing the patients for this challenging treatment procedure.

Despite problems in recruitment, our prehabilitation concept was safe, feasible and effective for the included patients. Our attrition rate was 87.5% and adherence was with 92% to HIIT and 85.4% to RE relatively high in comparison to two pilot studies prior autologous and allo-HCT^22,35^ and to our RCTs in allo-HCT during and after transplantation^36,37^. In comparison to the concept of Wood et al. our intervention program was supervised, more standardised in terms of the exercise contend (free chosen activity vs HIIT protocol on a bicycle ergometer of 8 × 1 min high-intensity at 90–100% PPO) and included moderate to high-intensity resistance exercise^34^. A recent review of HIIT in cancer patients reported an adherence between 71.2 and 95.6%^38^. We think that the combination of short intervention period, supervised training (including appointments with the exercise facility) and diverse exercise content have led to the high adherence rate in our study. The very good feedback on likeability, helpfulness and enjoyment underline the feasibility of the exercise program. However, for future studies it would be helpful to have additionally an exercise facility integrated in the transplant unit so that patients could also train directly in the clinic. Particularly in the view of the limited time available, this would make the implementation of exercise much easier.

In our study, only 11 sessions (6.3%) were rated with “overstraining” indicating that our demanding high to moderate intensity exercise program was feasible and acceptable. However, four exercise sessions had a premature ending because of exhaustion and lack of energy and in five sessions, the intensity needed to be reduced or single exercises (RE) needed to be terminated. Since patient’s prior allo-HCT have already limitations in physical capacity caused by previous treatments^14^ and the clinical status can vary, a good monitoring of training load during every exercise session is mandatory.

Evidence showed that the objectively measured physical capacity prior allo-HCT has an impact on outcomes, such as prognosis and symptom severity^11,15–18^. Therefore, it is of high clinical importance to increase CRF and functionality already prior allo-HCT. Furthermore, it is assumed that building up physiological reserve will lead to better treatment tolerance due to higher level of physiologic function throughout treatment and may mitigate further decline after allo-HCT due to complications e.g. GvHD. GvHD after allo-HCT is associated with a further decline in functional performance and reduced QOL^39^. Despite the short intervention period of 5.5 weeks available and 9.5 exercise sessions performed, we observe significant effects on physical capacity measures.

VO2peak increased significantly from 20.4 to 23.4 ml/kg/min and PPO increased significantly from 117 to 131 W. This VO2 peak improvement correspond to a very large effect (Choen’s d = 1.6). However, these results are in line with the results of another pilot study prior transplantation, where 3.7 ml/kg/min of VO2peak improvement in the allo-HCT patients could be observed^22^. It could be shown that a VO2peak decrease of 1 ml/kg/min was associated with higher mortality rates after allo-HCT (HR 1.13)^17^. Interestingly, there was a large variance in the initial performance level and the response of the patients seemed not to depend on number of exercise sessions performed or initial value. However, in 5 patients the VO2peak value prior allo-HCT lay below the threshold of 18 ml/kg/min, which was proposed to be a threshold for physically independence in older adults^40^. Though, after intervention only 2 patients lay below the threshold. Interestingly, these 2 patients were the ones who did not improve their VO2peak but deteriorated. We can speculate, if for those two patients the intervention program did not fit. However, another patient that started on a very low level could clearly increase his VO2peak, but had more exercise sessions available. Therefore, it could be that patients with a very low performance need more sessions to achieve a clear training effect on VO2peak.

We can speculate on further influencing factors that may play a role for the response. For example, clinical factors, e.g. the pre-treatment including cardiotoxic argents, blood counts (e.g. hemoglobin value) may play a role here^41^. In or previous work we performed an analysis on influencing clinical factors on the VO2peak prior allo-HCT^14^, however, it would be interesting to perform an subgroup analysis on the influence of different factors on the exercise response prior allo-HCT in future studies.

The 6-min walking distance improved significantly by 30.5 m which is comparable to other studies in this setting^22^. In clinical populations, the minimum clinically important difference is defined as 14.0–30.5 m^42^. In allo-HCT it could be shown that each 100 m decrease was associated with a higher risk of mortality (HR 2.98)^17^. In our previous RCT we demonstrated that a higher baseline fitness (meters in 6 min walking test) were associated with lower total mortality rates in a multivariate model^18^. This study could also demonstrate that a partly supervised exercise program may reduce mortality rates after allo-HCT^18^.

Since not only a great CRF seems to improve outcome after allo-HCT, but also sarcopenia was associated with higher non-relapse mortality and shorter overall survival after allo-HCT^15,43^, we included RE in our exercise intervention. Our previous RCTs in patients during and after allo-HCT demonstrated the feasibility and effectiveness of combined interventions^18,36,37^. A recent study revealed the HGS as a significant predictor for non-relapse mortality (HR 2.56)^44^. Our results showed a significant improvement of HGS of about 2 kg, indicating a positive effect of our RE on HGS.

So far, very few prehabilitation studies focused on PROs. However, not only physical capacity but also a better QOL prior allo-HCT was associated with reduced risk of mortality^45^. A reduced symptom severity, e.g. reduced fatigue and physical functioning will lead to better general QOL throughout treatment. Our intervention had a significant positive impact on physical functioning, role functioning, emotional functioning, fatigue (physical fatigue, reduced activity) and dyspnoea. Previous interventions with moderate intensity training during or after allo-HCT demonstrated the positive effect of exercise on various PROs^9^.

Strengths of our study included a sound intervention concept, including highly standardised HIIT and RE and individualized exercise prescription using percentages from the individual maximal capacity for both, HIIT and RE. Additionally, a highly standardised measurement protocol was applied, including gold-standard methods to assess CRF. Furthermore, our exercise intervention was supervised to ensure a safe completion and good adherence to the exercise protocol. Our study also used a multicentre approach to gain results regarding recruiting structures from different study centres. However, our study has several limitations and our findings must be interpreted cautiously. First of all, we had a small sample size due to a low recruitment rate and most of the participants were being recruited in one study centre (Heidelberg). Furthermore, our sample seems not to fully represent patient’s diversity according disease distribution prior allo-HCT because most participants were diagnosed with MDS or Lymphoma. Normally, AML is the most frequently diagnosis for allo-HCT. Finally, this was a single arm pilot study with a small sample size and larger randomized studies are necessary to confirm our results.

## Conclusion

Our study showed that HIIT and high-to moderate RE is feasible for patients prior allo-HCT and may improve CRF and PROs in a very short timeframe. This is clinically highly relevant since a higher physical capacity prior allo-HCT may improve symptoms and survival. However, recruitment rates were much too low and recruitment needed to be terminated. Therefore, we conclude that the time-point in the weeks prior allo-HCT need to be expanded and include also patients with planned allo-HCT but without further scheduling. Especially patients with high-risk profiles and low physical capacity should be offered a structured and individually tailored exercise program as supportive care option to enhance physical capacity/CRF and ultimately improve outcomes such as survival.

## Data Availability

The datasets generated during and/or analyzed during the current study are available from the corresponding author on a reasonable request.
